# Associations of linear growth with body composition of perinatally HIV-infected African adolescents

**DOI:** 10.1017/S000711452400134X

**Published:** 2024-12-28

**Authors:** Suzanne Filteau, Victoria Simms, Molly Chisenga, Cynthia Kahari, Nyasha Dzavakwa, Cassandra Namukonda, Kate A. Ward, Lackson Kasonka, Celia L. Gregson, Jonathan Wells

**Affiliations:** 1 Faculty of Epidemiology and Population Health, London School of Hygiene and Tropical Medicine, London, UK; 2 University Teaching Hospital, Lusaka, Zambia; 3 The Health Research Unit – Zimbabwe (THRU-Zim), Biomedical Research and Training Institute, Harare, Zimbabwe; 4 MRC Lifecourse Epidemiology Unit, University of Southampton, Southampton, UK; 5 Musculoskeletal Research Unit, Bristol Medical School, University of Bristol, Bristol, UK; 6 Institute of Child Health, University College London, London, UK

**Keywords:** Stunting, Body composition, HIV, Adolescent, Africa

## Abstract

The prevalence of poor linear growth among African children with perinatally acquired HIV remains high. There is concern that poor linear growth may to lead to later total and central fat deposition and associated non-communicable disease risks. We investigated associations between height-for-age *Z* score (HAZ) and total and regional fat and lean mass measured by dual-energy X-ray absorptiometry, expressed as internal population *Z* scores, among 839 Zimbabwean and Zambian perinatally HIV-infected male and female adolescents aged 11–19 years. Stunting (HAZ < –2) was present in 37 % of males and 23 % of females. HAZ was strongly positively associated with total, trunk, arm and leg fat mass and lean mass *Z* scores, in analyses controlling for pubertal stage, socio-economic status and HIV viral load. Associations of linear growth with lean mass were stronger than those with fat outcomes; associations with total and regional fat were similar, indicating no preferential central fat deposition. There was no evidence that age of starting antiretroviral therapy was associated with HAZ or body composition. Non-suppressed HIV viral load was associated with lower lean but not fat mass. The results do not support the hypothesis that poor linear growth or stunting are risk factors for later total or central fat deposition. Rather, increased linear growth primarily benefits lean mass but also promotes fat mass, both consistent with larger body size. Nutritional and/or HIV infection control programmes need to address the high prevalence of stunting among perinatally HIV-infected children in order to mitigate constraints on the accretion of lean and fat mass.

Children with perinatally acquired HIV have multiple co-morbidities despite antiretroviral therapy (ART)^(1)^. Poor linear growth during early childhood is common among African children with perinatally acquired HIV^(2,3)^. The effectiveness of ART in improving linear growth may depend on the age of ART initiation and on whether the child lives in a high-income or resource-constrained setting^(3)^. The situation in Africa may be improving with the roll-out of routine testing of HIV-exposed infants and early initiation of ART^(4)^.

Although poor linear growth or stunting (low length/height-for-age *Z* score (HAZ)) is associated with many adverse health outcomes^(5)^, there is mixed evidence as to whether growth recovery during childhood, in terms of either height or weight, might actually be harmful. The concern is that rapid growth may lead to increased total or central fat deposition and hence increased risk of chronic metabolic diseases. There is evidence both for^(6)^ and against^(7,8)^ the hypothesis of increased risk of excess fat among people with slow early linear growth. The difference could be partly due to the measure used for excess fat since presenting it as a percent of weight^(6)^ could result in higher values due to the lower amounts of lean mass in stunted children. Differences also likely depend in part on whether the diet and environment, including prevalence of infections, permitted later catch-up growth among stunted children or whether these factors remain similar to those which caused stunting in the first place. A diet of excess energy content, often deficient in micronutrients, can lead to fat deposition, and inflammation may result in deposition of fat rather than lean tissue^(9,10)^. In lowland Nepal, in an environment of long-term nutritional stress, children with stunting in early life maintained substantially lower levels of body fat in mid-childhood^(8)^. This raises the question as to what would happen if the nutritional stress was relieved among such children. Provision of a nutritionally complete diet may permit deposition of lean rather than fat mass: a recent trial of lipid nutritional supplements to already stunted Ugandan children aged 12–59 months found that lipid nutritional supplements increased children’s HAZ and weight and that most weight gain was fat-free mass, comprising lean, mainly muscle and bone^(11)^. In addition, timing of the anthropometric gains appears important, though here too the data are inconsistent. More rapid linear growth after, but not before, age 5 years has been associated with increased percent body fat, blood pressure and insulin resistance in Indian children aged 13·5 years^(12)^. In Ethiopian children aged 5 years, weight gain velocity between 4 and 5 years had larger associations with fat mass and markers of chronic disease risk than did weight gain at earlier periods from birth^(13)^. This study, which had detailed body composition data over time, also found increases in fat-free mass with later growth, illustrating the problems of using BMI which does not differentiate fat mass from fat-free mass, as the only marker of adiposity when investigating chronic disease risk. The multi-country COHORTS collaboration found that conditional relative weight in both early childhood and adolescence, but not in infancy, was associated with increased fat mass relative to fat-free mass in adulthood^(14)^. Finally, stunted children living with HIV and with access to sufficient energy content may be at particular risk of depositing fat mass due to ongoing inflammation in many HIV-infected people in spite of ART treatment^(15)^. In adults with HIV, ongoing inflammation or raised plasma HIV viral load were associated with preferential deposition of fat rather than lean mass^(9,10)^.

It is important to determine whether early linear growth, possibly affected by ART, of perinatally HIV-infected children is associated with their body composition and therefore potential risk of later chronic metabolic diseases. We wished to test the hypothesis that linear growth is associated with total and regional body fat and lean mass in adolescents with perinatal HIV infection and a high prevalence of stunting.

## Methods

### Study design and participants

The study used baseline data from the vitamin D_3_ and calcium carbonate supplementation for adolescents with HIV to reduce musculoskeletal morbidity and immunopathology (VITALITY) trial, a randomised controlled trial conducted in Zambia and Zimbabwe (Pan African Clinical Trials Registry ID: PACTR20200989766029). The trial primary outcome is total body less-head bone mineral density *Z* score.

Participants in Lusaka were identified from urban outpatient HIV clinics and referred for recruitment at the Women and New-born Hospital of the University Teaching Hospitals, and in Harare, at the Children’s Hospital at Sally Mugabe Central Hospital. Inclusion criteria were age 11–19 years, perinatally acquired HIV infection, taking ART for at least 6 months, having a firm home address and intending to remain there for 96 weeks, having a defined caregiver (for those aged <18 years), being aware of their HIV status (for those aged >12 years), willing to give blood samples and rectal swabs, guardian consent and participant assent for those aged <18 years or participant consent for those aged ≥18 years. Exclusion criteria were any condition likely to prove fatal during the study period, taking tuberculosis treatment, being pregnant, having a condition likely to lead to lack of understanding of or cooperation with study procedures, history of thyrotoxicosis, lymphoma, renal calculi, chronic renal disease, osteomalacia, hypercalcemia or a disorder of phosphate metabolism, physical or radiological signs of rickets or osteomalacia, living in the same household as a trial participant, and having a high likelihood of non-adherence to trial medication. Further details about the VITALITY trial protocol are published^(16)^.

### Anthropometry

Height and weight were measured in triplicate using standard methods^(17)^. The range of the three measures was checked to look for outliers which were corrected or removed when found. Means of the remaining valid data were used in analyses. Technical error of measurement calculations were performed periodically, and agreement among anthropometrists was within acceptable limits^(18)^. BMI was calculated as weight in kg divided by height in m^2^. Anthropometric *Z* scores for HAZ and BMI-for-age *Z* score were calculated using the Stata zanthro commands and the UK reference values (Stata 18, College Station). The UK growth references were used for consistency in the VITALITY study since the references for bone density by dual-energy X-ray absorptiometry (DXA), the primary outcome in the trial, are UK references^(19)^. The UK growth references have the added advantage of going up to 20 years^(20)^, so we could use the same reference for all participants. Stunting was considered HAZ <–2. BMI categories were determined from BMI-for-age *Z* score for participants under 18 years: <–3, grade 3 thinness; –3 to <–2, grade 2 thinness; –2 to <–1, grade 1 thinness; –1 to +1, normal; > = +1, overweight or obese. For participants over 18 years, adult BMI categories were used: < 16 kg/m^2^, grade 3 thinness; 16 to < 17 kg/m^2^, grade 2 thinness; 17 to <18·5 kg/m^2^, grade 1 thinness; 18·5–<25·0 kg/m^2^, normal; > = 25·0 kg/m^2^, overweight or obese.

### Body composition

The study outcomes were body fat and lean (not including bone) masses assessed by DXA. Different DXA machines were available at the two sites: Hologic QDR DXA (Hologic) with Apex software version 4.5 in Zambia and GE Lunar iDXA (GE Healthcare) with ENCORE software version 18 in Zimbabwe. For each of the machines, daily calibration was done with the manufacturer-provided spine phantom. The European spine phantom was used for cross-calibration of the DXA machines. At both sites, DXA scans were repeated in sixty participants to assess reproducibility and the % CV were <2 % (lean mass) and <3 % (fat mass). The different manufacturer models differ in technology and assumptions made to estimate body composition^(21)^. Because of the different DXA machines, we controlled for country in all analyses. We considered as outcomes total, trunk, arm, and leg fat and lean mass. Further details of the DXA methods are in online Supplementary Material.

### Blood sampling and analysis

Fasting venous blood samples were collected, and plasma HIV viral load was measured using the Hologic Panther and GeneXpert machines in Zambia and the Roche COBAS Ampliprep/COBAS Taqman48 in Zimbabwe.

### Ethical approval

This study was conducted according to the guidelines laid down in the Declaration of Helsinki, and all procedures involving human participants were approved by the University of Zambia Biomedical Research Ethics Committee (1116-2020), the Medical Research Council of Zimbabwe (A/2626), the Biomedical Research and Training Institute Internal Review Board (Zimbabwe, AP158/2020)), the Ethics Committee of Harare Central Hospital (HCHEC 030320/12), and the Ethics Committee of the London School of Hygiene and Tropical Medicine (22030). Participants over 18 years provided written informed consent. For those under 18 years, their guardian provided written informed consent and the participant provided assent.

### Data management, sample size and statistical analyses

Data were collected on tablets using electronic forms designed with OpenDataKit (ODK) software. Further cleaning and checking of distributions as well as analyses were done using Stata 18 (Stata Corp). Socio-economic status (SES) was categorised by quintiles of the first component in a principal component analysis of a list of assets potentially owned by participants’ families^(22)^.

The sample size available was based on the primary VITALITY outcome. In the available sample of 393 males, the study had 80 % power to detect an effect size of 0·02 and 90 % power to detect an effect size of 0·027 using multivariable linear regression. In the sample of 446 females, the minimum detectable effect sizes were 0·017 at 80 % and 0·023 at 90 %.

Because the UK DXA body composition reference may not be generalisable to perinatally HIV-infected African children, we calculated internal population *Z* scores using the LMS method^(23)^ for DXA data. This method generates three parameters (LMS for power, mean and standard deviation) for each month age band, allowing individual *Z* scores to be calculated using these age- and sex-specific parameters and the individuals’ measurements.

We first investigated covariables which might need to be controlled for in analyses of body composition: pubertal stage, age of starting ART, plasma HIV viral load and SES. Age of starting ART was based on clinic records held by participants. We analysed age of starting ART both as a continuous variable, using linear regression to compare it with anthropometric and body composition outcomes, and as a categorical variable divided as <2 years, 2–5 years and >5 years. For puberty, we used the five Tanner stages, based on observation by clinic staff, testes size for males and breast size for females. Associations with Tanner stage were by ANOVA. Because of evidence that high viral load or inflammation may be associated with fat, rather than lean, mass deposition^(9,10)^, we investigated whether viral load was associated with body composition. Viral load was categorised as <60 copies/μl which was the detection limit in Lusaka, 60–1000 copies/μl, or >1000 copies/μl.

Since there is evidence that most stunting occurs before age 2 years after which it is hard to reverse^(24)^, we considered that HAZ, when measured in adolescence, reflected mainly early childhood faltering in linear growth. Thus, HAZ was considered the exposure. The outcomes were internal age- and sex-adjusted *Z* scores for total and regional fat and lean mass by DXA. As the two sexes have well-established different patterns of fat and lean tissue accretion during adolescence, results are presented separately by sex. Analyses were controlled for country. Other covariables considered were current age, age of starting ART, pubertal stage, viral load and SES quintile. As exploratory analyses, conducted after seeing the figures showing associations between linear growth and body composition, we plotted regression residuals against HAZ to visualise whether variability differed at different levels of HAZ (i.e. heteroskedasticity). We also investigated, using piecewise regression, whether the regression coefficients differed above and below HAZ of –2, that is, the cut-off for stunting. Finally, to check if there was preferential central fat deposition, we analysed trunk fat *Z* score against HAZ, controlling for the mean of arm and leg fat *Z* score, that is, peripheral fat.

## Results

A flow chart showing participants eligible and recruited is in online Supplementary Fig. 1. Of the 842 participants recruited, one was missing height data and two were missing DXA data, so they were excluded from analyses. Just over half of the adolescents in each country were female and the age distribution, as planned during recruitment, was approximately equal across age bands (Table 1). The mean (sd) age participants started ART, mostly tenofovir-based regimens, was 6·5 (sd 4·2) years with Zambians starting slightly later than Zimbabweans. A large proportion of adolescents in both countries presented with non-suppressed HIV viral loads. Only half the participants had both parents still living. Parental educational level was slightly higher in Zimbabwe than Zambia.

**Table 1.** Description of the study population of adolescents with perinatal HIV infection

	Zimbabwe (*N* 420)		Zambia (*N* 419)	
	#	% or sd	#	% or sd
Sex (#, % female)	217	52 %	229	55 %
Age group (years)				
11–13	89	21 %	100	24 %
13–15	73	17 %	110	26 %
15–17	97	23 %	90	21 %
17–20	161	38 %	119	28 %
Tanner stage, testes, males				
1	19	9 %	15	8 %
2	30	15 %	54	28 %
3	43	21 %	47	25 %
4	53	26 %	40	21 %
5	57	28 %	34	18 %
Tanner stage, breasts, females				
1	27	13 %	16	7 %
2	27	13 %	18	8 %
3	33	15 %	43	19 %
4	51	24 %	61	27 %
5	78	36 %	91	40 %
Age started ART,* years (mean, sd)	5·8	sd 4·0	7·2	sd 4·4
<2 years	70	17 %	67	16 %
2–<5 years	139	33 %	109	26 %
>=5 years	211	50 %	243	58 %
ART* regimen				
Tenofovir	326	78 %	357	85 %
Azidothymidine	35	8 %	50	12 %
Abacavir	59	14 %	11	3 %
HIV viral load (copies/μl)				
<60	320	76 %	354	84 %
60–1000	41	10 %	24	6 %
>=1000	59	14 %	41	10 %
Parental survival				
Both known alive	191	45 %	202	48 %
One parent known to be dead	152	36 %	144	34 %
Both parents known to be dead	68	16 %	60	14 %
Missing data	9	2 %	13	3 %
Mother’s highest education level				
None	4	1 %	20	5 %
Primary	34	8 %	131	31 %
Secondary	287	68 %	162	39 %
College/university	17	4 %	13	3 %
Missing	78	19 %	93	22 %
Father’s highest education level				
None	0	0 %	6	1 %
Primary	16	4 %	37	9 %
Secondary	266	63 %	150	36 %
College/university	25	6 %	53	13 %
Missing	113	27 %	173	41 %
Housing own home	188	45 %	183	44 %
Rent main dwelling	48	11 %	39	9 %
Rent or use part of dwelling	184	24 %	197	47 %

* ART, antiretroviral therapy.

Anthropometric *Z* scores based on the UK reference were similar in the two countries (Table 2). Males generally had greater anthropometric deficits in both linear growth and BMI, compared with the UK reference population, than females. About a third of males and a quarter of females were stunted. A higher proportion of males than females were undernourished based on BMI. Very few adolescents were overweight or obese.

**Table 2.** Anthropometric *Z* scores, using UK reference, of perinatally HIV-infected adolescent study participants*,†

	Zimbabwe				Zambia			
	Males (*n* 204)		Females (*n* 216)		Males (*n* 190)		Females (*n* 230)	
	Mean	sd	Mean	sd	Mean	sd	Mean	sd
Height-for-age *Z*	–1·70	1·09	–1·24	1·06	–1·66	1·02	–1·19	1·04
BMI *Z*	–1·01	0·96	–0·28	1·04	–1·35	0·97	–0·44	1·03
	#	%	#	%	#	%	#	%
Height-for-age *Z* < –2	75	37	49	23	70	37	54	24
BMI group‡								
Grade 2 thinness	24	11	10	5	36	19	15	7
Grade 1 thinness	45	22	30	14	59	31	37	16
Normal	135	67	159	73	94	49	167	73
Overweight or obese	0	0	18	8	1	0·5	10	4

* BMI.

† *Z* scores were calculated using zanthro in Stata and the UK growth reference.

‡ For participants >=18 years, BMI groups were as follows: < 16 kg/m^2^, grade 3 thinness; 16 to < 17 kg/m^2^, grade 2 thinness; 17 to <18.5 kg/m^2^, grade 1 thinness; 18.5–<25.0 kg/m^2^, normal; >= 25.0 kg/m^2^, overweight or obese. For participants <18 years, categories were based on BMI *Z* scores: <–3, grade 3 thinness; –3 to <–2, grade 2 thinness; –2 to <–1, grade 1 thinness; –1 to +1, normal; >= +1, overweight or obese.

In males, more mature pubertal stage was associated (*P*_for trend_ across Tanner stages <0·001) with increased lean mass internal *Z* score but not with HAZ, BMI-for-age *Z* score or total or regional fat *Z* scores (online Supplementary Fig. 2). In females, more mature pubertal stage was associated (*P*_for trend_ <0·001) with greater *Z* scores for all anthropometric and body composition variables analysed.

In both the continuous variable and categorical analyses, there was no evidence of an association between age of starting ART and any anthropometric or body composition outcome except leg fat *Z* (coefficient: 0·021, 95 % CI 0·005, 0·037, *P* = 0·01) (online Supplementary Fig. 3). Therefore, age of starting ART was not included as a covariable in further analyses.

Detectable or high plasma viral load (as categories) were associated with lower total and regional lean mass but were not associated with fat mass (online Supplementary Table 1). SES was associated with all markers of lean mass, although not always in continually increasing fashion, whereas it was not associated with fat mass; results for total fat and lean *Z* scores by SES are shown in online Supplementary Fig. 4.

**Fig. 1.**
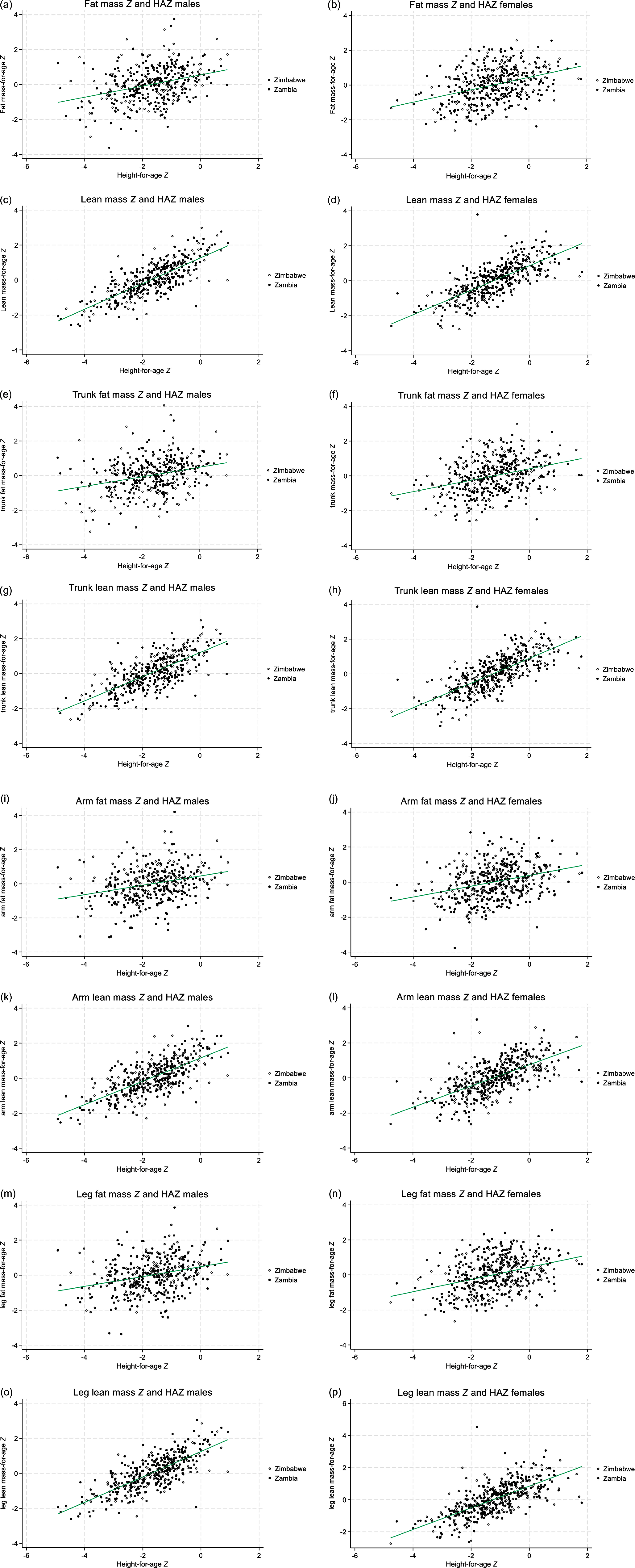
Scatter plots with line of best fit, describing the relationships between height-for-age *Z* (HAZ) score and lean and fat mass *Z* scores by body region, in males and female adolescents living with HIV. (a, b) Total fat mass *Z*, males, females; (c, d) total lean mass *Z* males, females; (e, f) trunk fat mass *Z*, males, females; (g, h) trunk lean mass *Z*, males, females; (I, J) arm fat mass *Z*, males, females; (k, l) arm lean mass *Z*, males, females; (m, n) leg fat *Z*, males, females; (o, p) leg lean mass *Z*, males, females. Body composition determined by dual X-ray absorptiometry and internal population *Z* scores used; HAZ is based on the UK reference.

Table 3 shows associations of total and regional fat *Z* scores with HAZ, controlling for country, Tanner stage, SES and viral load category; graphical representations are in Fig. 1. For all outcomes and for both sexes, greater HAZ was associated with greater total and regional fat and lean *Z* scores; regression coefficients were similar for total, central and peripheral fat mass *Z* scores and for total, central and peripheral lean mass *Z* scores. Associations of HAZ with lean mass *Z* scores were stronger than for fat mass *Z* scores. Adding peripheral fat *Z* score to the covariables when analysing trunk fat *Z* score reduced the association with HAZ, but this remained positive: the coefficient (95 % CI) for males was 0·07 (95 % CI 0·02, 0·11; *P* = 0·005) and for females was 0·04 (95 % –0·00, 0·07, *P* = 0·075). From plots of residuals against HAZ, there was no evidence of heteroskedasticity. Using piecewise regression, there was no evidence that the strength of the association between HAZ and lean mass differed above and below a HAZ of –2 (data not shown).

**Table 3.** Associations between height-for age *Z* score and total and regional fat *Z* scores from dual-energy X-ray absorptiometry (DXA), in males and female adolescents living with HIV*,†

	Males			Females		
Dependent variable	Coefficient	95 % CI	*P*	Coefficient	(95 % CI)	*P*
Total fat *Z*	0·29	0·21, 0·38	<0·001	0·30	0·23, 0·38	<0·001
Total lean *Z*	0·64	0·58, 0·69	<0·001	0·61	0·55, 0·66	<0·001
Trunk fat *Z*	0·28	0·19, 0·36	<0·001	0·28	0·20, 0·36	<0·001
Trunk lean *Z*	0·59	0·53, 0·65	<0·001	0·60	0·55, 0·66	<0·001
Arm fat *Z*	0·22	0·14, 0·30	<0·001	0·24	0·17, 0·32	<0·001
Arm lean *Z*	0·57	0·51, 0·63	<0·001	0·53	0·46, 0·59	<0·001
Leg fat *Z*	0·25	0·16, 0·33	<0·001	0·30	0·22, 0·37	<0·001
Leg lean *Z*	0·63	0·58, 0·69	<0·001	0·58	0·52, 0·64	<0·001

* The exposure, height-for-age *Z* score, is based on the UK reference, and the DXA body composition *Z* scores are internal population scores.

† Coefficients and *P* values are from linear regressions, controlling for country, Tanner stage, socio-economic quintile, and viral load (<60, 60–100, >1000 copies/μl). The coefficients represent *Z* score differences for 1 sd change in HAZ.

## Discussion

In this population with a high prevalence of stunting, HAZ, likely reflecting linear growth in early childhood, was positively associated with both fat and lean mass *Z* scores in adolescence. Associations with lean mass *Z* score were stronger than those for fat mass *Z* scores. Total, peripheral and central fat had similar associations with linear growth, and HAZ remained positively associated with trunk fat when peripheral fat was included in the regression, indicating no preferential deposition of central fat in adolescents with lower linear growth.

A few publications have suggested that people who experienced poor linear growth in early childhood are at risk for later increases in total or central fat^(6,25,26)^, or reduced fasting fat metabolism which could lead to obesity^(27)^. However, there are also many publications which do not show that people with poor linear growth are at increased risk of overweight, altered fat metabolism or chronic diseases^(7,8,12,28,29)^. The difference may be due to whether or not the child’s environment in later years permits high energy intake, often associated with poor micronutrient intake, and weight gain^(26)^. It is possible that the adolescents in our study did not deposit excess fat in spite of poor linear growth because they continued to live under the low socio-economic conditions which contributed to poor growth earlier in life. It could be that infection and inflammation were the dominant factors affecting growth and body composition of this HIV-infected population^(9,10)^. Although we controlled for SES quintile, it is possible that aspects of SES other than family assets, for example, parental education, might have been more important contributors to body composition. We did not include parental education in analyses because of the large amount of missing data, mainly due to children missing parents, but it was highly associated with SES (not shown).

A sizeable proportion of the study population had high viral load despite ART for several years. High viral load may be associated with inflammation, and inflammation has been associated with preferential deposition of fat over lean tissue^(9,10)^. Our results are in agreement with this other work since we showed that high viral load was associated with lower lean mass but no difference in fat mass. These results need to be considered in light of the fact that viral load can fluctuate over much shorter timescale than body composition. However, repeated episodes of infections or inflammation in these adolescents throughout their lives may have led to the stronger associations of HAZ with lean than with fat mass.

Although others have shown that starting ART at a younger age can improve growth of perinatally HIV-infected children^(3)^, we did not find this. A possible reason for this is that the adolescents in our study were born before early HIV testing of HIV-exposed infants became routine. Children may have been tested and started on ART only when they showed symptoms, which include growth faltering. Children diagnosed after showing symptoms at younger age may have been infected earlier, for example, *in utero* or at delivery; this period of the first 1000 d is known to be a critical period for child growth^(30)^.

We used internal age-adjusted *Z* scores for body composition measures, so there was no association of these with age. However, increasing Tanner stage, which was associated with age, was associated with increases in all body composition measures in females and in lean mass *Z* scores in males. This may reflect the known variation in the timing of puberty, the magnitude of the pubertal growth spurt, and body proportions and composition at the end of growth, all of which make it difficult to establish a universal growth reference for adolescents^(31)^.

Greater linear growth is expected to increase both lean mass and fat mass, for different reasons. Lean mass is closely associated with skeletal dimensions, since longer limbs equate to longer muscles and so must inevitably increase the associated muscle mass. Bones must also scale to this to ensure they are strong enough for their length, and larger muscles lead to increased bone mass and cross-sectional area. A longer trunk indicates larger total organ mass, though it remains unclear which specific organs are affected. A similar scenario is expected for fat mass. For a given subcutaneous skinfold thickness (cylinder thickness), a longer limb (cylinder length) translates into a greater mass of fat in the limb, and the same scenario applies to the trunk. On the basis that fat stores defend against deficits in energy supply (inadequate diet, raised energy costs during infection), then a taller person with greater lean mass and energy turnover requires a greater mass of fat to fund the energy shortfall for any given period of time. Our findings support both these predictions.

Strengths of the study include its large sample size of African adolescents and its detailed body composition analysis by DXA. In addition, the use of internal *Z* scores for body composition ensured that the results are relevant to the population in question, while the use of external, UK, references for the linear growth measures allowed comparison of the population with other cohorts. A limitation was that few of the adolescents were overweight, so we may not have had either the required range of body composition or adequate statistical power to investigate fully possible associations of body fat with linear growth. Most participants were on the same ART regimen, so we were unable to investigate whether there were differences by regimen. Although the study used two types of DXA scanning, there was good reproducibility, and we controlled for country to account for this. Finally, the study was cross-sectional with anthropometry, body composition and viral load measured at a single time point, so we cannot determine causality.

### Conclusion

While there remain reasons to be concerned about the high prevalence of poor linear growth amongst perinatally HIV-infected adolescents, they do not appear to be at risk of excess total or regional fat. They should be included with other adolescents in public health interventions promoting healthy diet and regular exercise in order to avoid increased risks of chronic diseases. In addition, HIV-infected adolescents should be monitored and supported to maintain a low viral load, since high viral load may decrease lean mass deposition. Further research should investigate cohorts of children who started ART in infancy after routine testing of children of HIV-infected mothers, since this may improve early growth whereas HIV testing usually after symptoms appeared was unable to do this. We show that the benefits of greater linear growth in this population are primarily in greater lean mass, a marker of metabolic health. In a population with very little overweight or obesity, and where low BMI remains more prevalent, the greater fat mass in taller children also indicates better health.

## Supporting information

Filteau et al. supplementary material Filteau et al. supplementary material

## Supplementary material

For supplementary material/s referred to in this article, please visit https://doi.org/10.1017/S000711452400134X
