# Supplementary material for: Associations of linear growth with body composition of perinatally HIV-infected African adolescents
Source: Br J Nutr. 2024 Nov 11;132(12):1593–601. doi: 10.1017/S000711452400134X (PMC11695111; doi:10.1017/S000711452400134X)
Supplement: Filteau et al. supplementary material [file S000711452400134Xsup001.docx]

**Associations of linear growth with body composition of perinatally HIV-infected African adolescents - Supplementary material**

Suzanne Filteau, Victoria Simms, Molly Chisenga, Cynthia Kahari, Nyasha Dzavakwa, Cassandra Namukonda, Kate A Ward, Lackson Kasonka, Celia L Gregson, Jonathan Wells

**Detailed DXA assessment methods**

Before undergoing the DXA scan, the participants were asked to confirm that they were able to lie flat on their back, that they had not had any radiology contrast enhanced examination within the past week and that they had removed all radiopaque objects, any clothing with metal fasteners or any metal jewellery. Within the DXA unit, each participant was asked to change into a loose comfortable fitting gown and to remove their shoes. For females, a urine pregnancy test on the day of the DXA scan was used to confirm they were not pregnant. For both the Hologic and GE Lunar machines, daily calibration was done with the manufacturer-provided lumbar spine phantom and a radiographic uniformity test was done once a week for the Hologic machine. Additionally, the European spine phantom (ESP, manufactured by QRM, Germany) was scanned at the beginning of the study before scanning any of the study participants and at regular intervals of approximately 6 months, throughout the period of the study to confirm no drift in measurement over the study period. At each of those times, the ESP was scanned 10 times on the same day. The same ESP was scanned on the Hologic DXA machine in Lusaka and on the GE Lunar iDXA machine in Harare. The same protocol was used for scanning the ESP at both sites. DXA scans were repeated in a subgroup of participants (n=60 at each site) to assess reproducibility. The repeat scans were performed two weeks from the initial scan and the coefficients of variation (% CVs) were <2% for lean mass and <3% for fat mass.

**Supplementary Table 1. Associations of plasma HIV viral load with total and regional fat Z scores from dual X-ray absorptiometry^1,2^**

|  | **Viral load category**  **(copies/μl)** | **Coefficient** | **95% CI** | **P** |
| --- | --- | --- | --- | --- |
| Total fat Z | 60-1000  >=1000 | -0.06  -0.06 | -0.32, 0.19  -0.27, 0.16 | 0.63  0.61 |
| Total fat-free Z | 60-1000  >=1000 | -0.27  -0.25 | -0.52, -0.01  -0.47, -0.04 | 0.04  0.02 |
| Total lean Z | 60-1000  >=1000 | -0.26  -0.25 | -0.52, -0.00  -0.47, -0.04 | 0.05  0.02 |
| Trunk fat Z | 60-1000  >=1000 | -0.02  -0.08 | -0.27, 0.24  -0.29, 0.13 | 0.91  0.45 |
| Trunk lean Z | 60-1000  >=1000 | -0.22  -0.20 | -0.48, 0.04  -0.41, 0.02 | 0.10  0.07 |
| Arm fat Z | 60-1000  >=1000 | -0.18  -0.02 | -0.43, 0.06  -0.23, 0.18 | 0.15  0.83 |
| Arm lean Z | 60-1000  >=1000 | -0.25  -0.30 | -0.50, 0.01  -0.51, -0.09 | 0.06  0.006 |
| Leg fat Z | 60-1000  >=1000 | -0.09  -0.01 | -0.34, 0.17  -0.22, 0.20 | 0.50  0.90 |
| Leg lean Z | 60-1000  >=1000 | -0.30  -0.29 | -0.56, -0.05  -0.51, -0.08 | 0.02  0.007 |

^1^ Coefficients and P values are from linear regressions, controlling for sex and country. Reference groups are those with viral load < 60 copies/ml.

**Supplementary figure legends**

**Supplementary Figure 1. Trial recruitment flow chart**

**Supplementary Figure 2. Pubertal stage and anthropometric and body composition Z scores**

^1^ BMIZ, body mass index-for-age Z score; HAZ, height-for-age Z score; DXA, dual X-ray absorptiometry

^2^ HAZ and BMIZ are based on UK reference values; fat and lean mass Z are internal to the population

^3^ Tanner stage for males determined by testes development; numbers at each stage were: stage 1, 34; stage 2, 84; stage 3, 90; stage 4, 93; stage 5, 91

^4^ Tanner stage for females determined by breast development; numbers at each stage were: stage 1, 43; stage 2, 45; stage 3, 76; stage 4, 113; stage 5, 170

^5^ For clarity, error bars are not shown but standard deviations were near 1.0 for all points.

^6^ For all measures for females and for lean, but not fat, mass for males, P<0.001 for trend across increasing Tanner stage.

**Supplementary Figure 3. Anthropometric and body composition Z scores and age of starting antiretroviral therapy**

^1^ ART, antiretroviral therapy; BMI, body mass index; DXA, dual X-ray absorptiometry

^2^ A) height-for-age Z, UK reference; B) body mass index-for-age Z, UK reference; C) total fat mass by DXA, internal Z scores; D) total lean mass by DXA Z, internal Z scores;

^3^ No correlations, controlling for sex, were statistically significant except for leg fat Z scores, P=0.01, and only in continuous analysis of ART starting age.

**Supplementary Figure 4. Socioeconomic quintile and total fat and lean mass Z scores**

Socioeconomic quintile was associated with total lean mass Z score, P=0.006, but not with total fat mass Z score, P=0.45.

**Supplementary Figure 1. Trial recruitment flow chart**

Screened

(age 11-19)

1432

Eligible

860 (60.0%)

Consented

845

Enrolled

842 (58.8%)

Refused 13

Unable to understand and give informed consent to trial 2

Not eligible

No defined guardian 188

On ART < 6 months 98

No fixed address 66

HIV diagnosis not disclosed to child 52

Not perinatal HIV 24

Sibling enrolled 42

Current TB or TB treatment 60

Pregnant/breastfeeding 24

Not living in catchment area 5

Adherence problems 5

Other exclusion criteria 8

Too sick to enrol 1

Unknown 2

**Supplementary Figure 2. Pubertal stage and anthropometric and body composition Z scores**

**
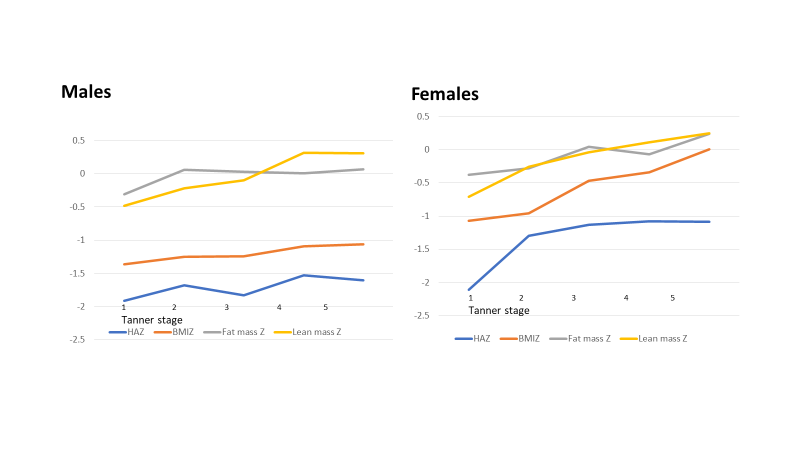
**

**Supplementary Figure 3. Anthropometric and body composition Z scores and age of starting antiretroviral therapy (ART)**

**A B**

**C D**

**Supplementary Figure 4. Socioeconomic quintile and total fat and lean mass Z scores**

**
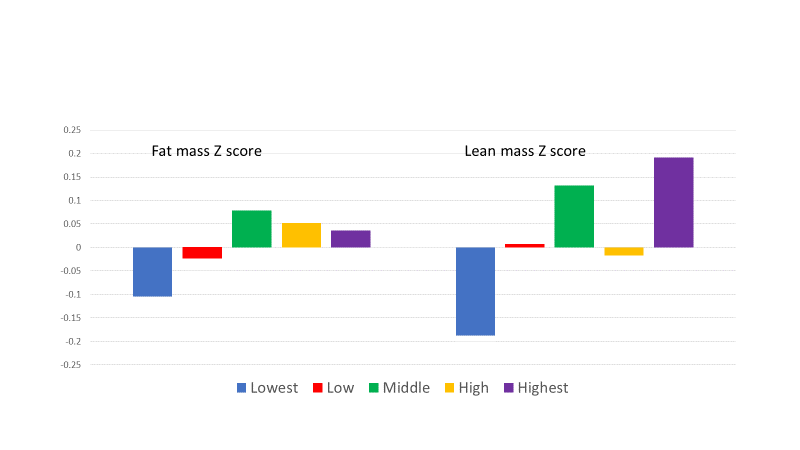
**
